# An unconventional case: endoscopic removal of a migrated intrauterine device perforating the rectum

**DOI:** 10.1055/a-2545-2511

**Published:** 2025-05-06

**Authors:** Xusheng Nie, Jia Liu, Jia Chen, Yi Luo, Deshan Xiong, Chaoqiang Fan, Cheng Liu

**Affiliations:** 1 Department of Gastroenterology, Yunyang People’s Hospital, Chongqing, China; 2 Department of Traditional Chinese Medicine, Xinqiao Hospital, The Army Military Medical University, Chongqing, China; 3 Department of Gastroenterology, Xinqiao Hospital, The Army Military Medical University, Chongqing, China

A 34-year-old woman was admitted to our hospital for removal of a migrated intrauterine device (IUD). The IUD had been inserted for about four years, and the patient had become pregnant twice without delivery. Ten months ago, the IUD was found to have migrated into the rectum before an extracorporeal shock wave lithotripsy in another hospital due to urinary stones. The patient was asymptomatic and did not undergo further treatment for personal reasons.

On arrival at our hospital, the patient’s abdomen was soft and non-tender. A little yellowish vaginal discharge was found. The cervix was enlarged and movable, without shaking pain or bleeding. The uterus was normal in size, movable, with no obvious pressure pain. A computed tomography (CT) scan of the abdomen was performed ( Fig. 1 ), which showed an inverted V-shaped metallic shadow in the pelvis. The ultrasound colonoscopy revealed penetration of the entire rectal wall by the IUD ( Fig. 2 ).

**Fig. 1:**
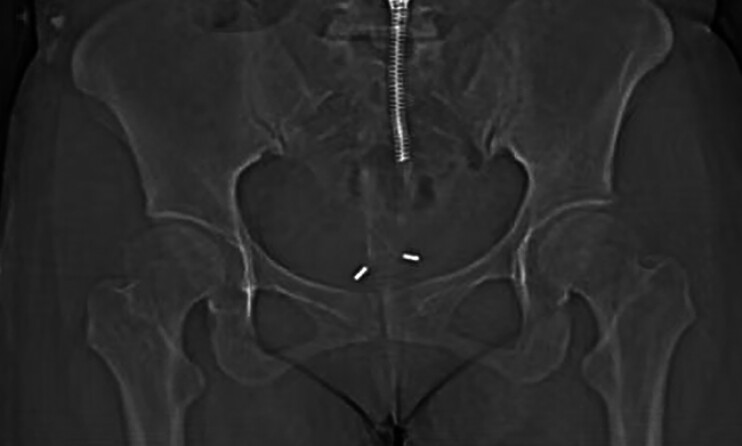
Coronal image of abdominal computed tomography scan indicates an inverted V-shaped metallic shadow in the pelvis.

**Fig. 2:**
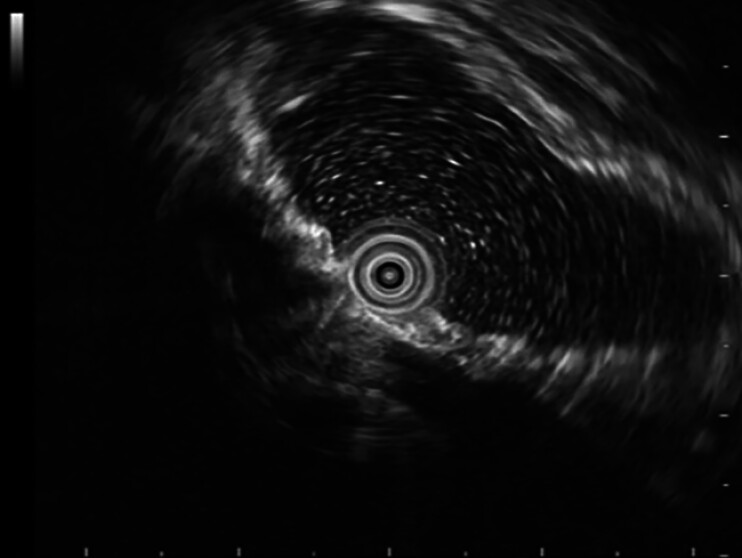
The ultrasound colonoscopy (20 MHz) revealed penetration of the entire rectal wall by the intrauterine device (IUD).

IUD perforations of the colon mostly cause chronic abdominal pain, vaginal bleeding, etc., and most perforations are of the sigmoid colon 1 . It was rare that our patient was asymptomatic and suffered no complications from the migrated IUD perforating the rectum. For IUDs that perforate the colorectum, laparoscopy and laparotomy are mostly reported to remove them 1 . However, endoscopic removal may be considered the preferred option 2 . Removal through the endoscopic route can effectively prevent surgical damage and facilitate the patient's recovery 3 . It is particularly efficient to pull out the IUD through the endoscope if the material is soft 3 . In our case, however, submucosal tunneling endoscopic resection (STER) 4 was successfully performed ( Video 1 ), and we were able to observe the whole arm of V-shaped metal piece directly and free it ( Fig. 3 , Fig. 4 ). It may be safer than cutting through the mucosa without directly seeing the IUD 5 . One year later, repeated colonoscopy showed white scar formation in the rectum without adverse effects ( Fig. 5 ).

Endoscopic removal of a migrated intrauterine device that perforated the rectum.Video 1

**Fig. 3:**
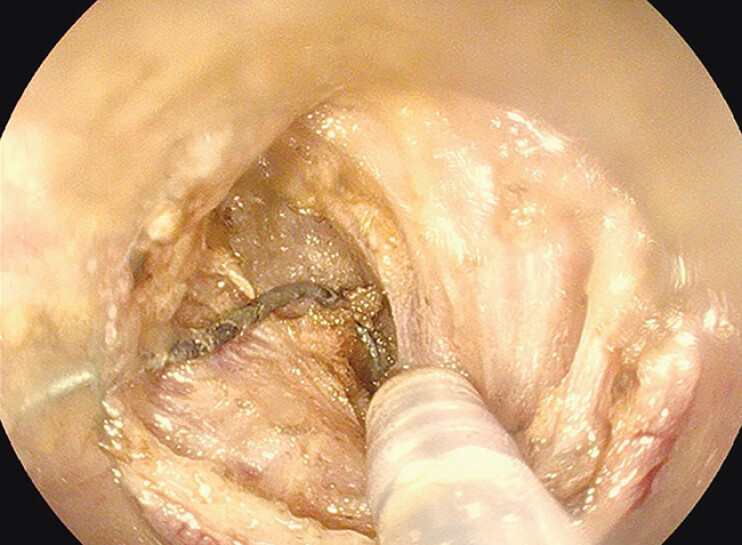
The deepest part of IUD was located at the peritoneal reflection line.

**Fig. 4:**
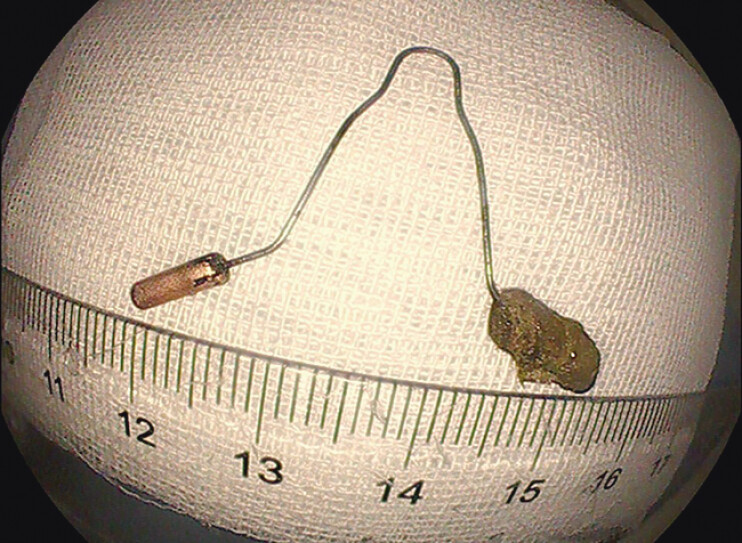
The V-shaped IUD that was removed during the operation.

**Fig. 5:**
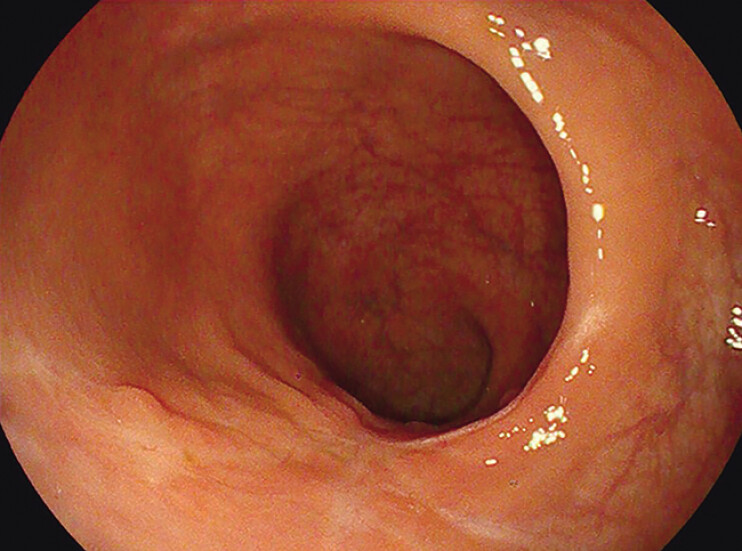
One year later, a repeat colonoscopy revealed good recovery of the rectum.

Endoscopy_UCTN_Code_TTT_1AQ_2AH

## Notes

Endoscopy E-Videos https://eref.thieme.de/e-videos
*E-Videos* is an open access online section of the journal *Endoscopy* , reporting on interesting cases and new techniques in gastroenterological endoscopy. All papers include a high-quality video and are published with a Creative Commons CC-BY license. Endoscopy E-Videos qualify for HINARI discounts and waivers and eligibility is automatically checked during the submission process. We grant 100% waivers to articles whose corresponding authors are based in Group A countries and 50% waivers to those who are based in Group B countries as classified by Research4Life (see: https://www.research4life.org/access/eligibility/ ). This section has its own submission website at https://mc.manuscriptcentral.com/e-videos .
